# Vaping causes an acute BMI‐dependent change in pulmonary blood flow

**DOI:** 10.14814/phy2.70094

**Published:** 2024-10-18

**Authors:** K. S. Burrowes, M. Seal, L. Noorababaee, B. Pontré, D. Dubowitz, R. C. Sá, G. K. Prisk

**Affiliations:** ^1^ Auckland Bioengineering Institute University of Auckland Auckland New Zealand; ^2^ Department of Anatomy and Imaging University of Auckland Auckland New Zealand; ^3^ Centre for Advanced MRI Auckland New Zealand; ^4^ Department of Medicine University of California San Diego California USA

**Keywords:** E‐cigarettes, MRI, perfusion, vaping, ventilation

## Abstract

Vaping use has skyrocketed especially among young adults, however there is no consensus on how vaping impacts the lungs. We aimed to determine whether there were changes in lung function acutely after a standard vaping session or if there were differences in lung function metrics between a healthy never‐vaping cohort (*N* = 6; 27.3 ± 3.0 years) and a young asymptomatic vaping cohort (*N* = 14; 26.4 ± 8.0 years) indicating chronic changes. Pulmonary function measurements and impulse oscillometry were obtained on all participants. Oxygen‐enhanced and Arterial Spin Labelling MRI were used to measure specific ventilation and perfusion, respectively, before and after vaping, and in the control cohort at baseline. MRI metrics did not show any significant differences in specific ventilation or perfusion after vaping. Heart rate increased post‐vaping (68.1 ± 10.5 to 71.3 ± 8.7, *p* = 0.020); however, this and other metrics did not show a nicotine dose‐dependent effect. There was a significant negative correlation between BMI and change in mean perfusion post‐vaping (*p* = 0.003); those with normal/low BMI showing an increase in perfusion and vice versa for high BMI. This may be due to subjects lying supine during vaping inhalation. Pulmonary function metrics indicative of airways resistance showed significant differences between the vaping and control cohorts indicating early airway changes.

## Graphical abstract

The effect of vaping on the lungs is not yet well understood; previous studies have shown mixed results. We applied functional MRI methods to measure regional ventilation and perfusion in the lungs before and after vaping. Results showed a strong correlation between BMI and the change in perfusion after vaping. Measurements of airways resistance showed significant differences between the vaping and control cohorts indicating early airway changes.

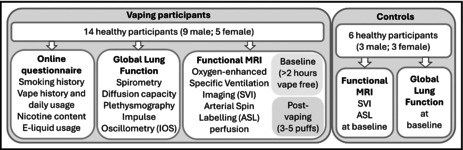

## 1 INTRODUCTION

Electronic cigarettes (ECs) or ‘vapes’ are a type of electronic nicotine delivery system that use heat to aerosolise a liquid (‘e‐liquid’) that the user inhales. ECs have been welcomed as a revolutionary tool to reduce the enormous harm caused by conventional cigarettes. However, there has been unprecedented uptake by adolescents and young adults around the world, many of them never‐smokers. For example, in New Zealand 15% of 15–17‐year‐olds and 25% of 18–24‐year‐olds reported vaping daily (Edwards et al., n.d.). Within the 18–24 year old age bracket, 37% reported to be never‐smokers and in those aged 15–17 years old the proportion of never‐smokers was 76% (Ministry of Health, 2023). Despite the popularity and social normalization of ECs, there are significant gaps in our understanding of how safe EC use is.

It is known that aerosolised e‐liquid contains toxicants, including some that are carcinogens and heavy metals (Burstyn, 2014; Chen et al., 2017; Farsalinos & Gillman, 2017; Kosmider et al., 2014). Mounting evidence has demonstrated that vaping causes inflammation and lung injury (Chatterjee et al., 2019; Davis et al., 2022; Muthumalage et al., 2019; Park et al., 2022; Sharma et al., 2021; Tsai et al., 2020), including in some cases a severe form of lung injury that has been termed ‘e‐cigarette and vaping‐associated lung injury’ (EVALI) (Marrocco et al., 2022). It is well known from studies of conventional smoking that inflammation is a major contributing factor to the development of tissue damage leading to chronic obstructive pulmonary disease (COPD) (Tetley, 2005). Asthma is another chronic inflammatory disorder of the lungs associated with airway narrowing and remodeling. One contributing factor to the development of asthma, particularly in young adults, is via exposure to inhaled toxicants. Recent studies have demonstrated some overlap between vaping and asthma, with EC use associated with earlier age of asthma onset in adults (Pérez et al., 2024), an association between EC use and asthma symptoms (Roh et al., 2023), and an overlap in sputum proteotypes in EC users and asthmatics (Hickman et al., 2024). These findings link EC use with asthma or an asthma‐like phenotype. Effects on the cardiovascular system have also been demonstrated in vaping (Espinoza‐Derout et al., 2022; Mohammadi et al., 2022; Nabavizadeh et al., 2022; Tattersall et al., 2023; Tsai et al., 2020). Cardiovascular measurements have shown that acute EC exposure increases blood pressure and heart rate (Tattersall et al., 2023), with prolonged elevations in blood pressure being linked with future endothelial cell dysfunction, a risk factor for cardiovascular disease (Totoń‐Żurańska et al., 2024). Long‐term EC exposure has been shown to increase blood vessel stiffness and cause endothelial cell damage (Tsai et al., 2020).

Magnetic Resonance Imaging (MRI) has been used previously to measure regional lung function to identify acute or chronic changes due to vaping (Kizhakke Puliyakote et al., 2021; Nyilas et al., 2022). Kizhakke Puliyakote et al. (2021) demonstrated an increased mismatch between ventilation and perfusion, a sign of reduced gas exchange efficiency, in nine vaping participants when compared to non‐vaping controls, which was exacerbated immediately after vaping. They showed an increase in ventilation heterogeneity and a decrease in perfusion heterogeneity immediately after vaping. Nyilas et al. (2022) used MRI to quantify functional impairment in ventilation and perfusion in 13 vaping volunteers before and immediately after vaping. They found an increase in total perfusion (assessed as a reduction in perfusion defects) immediately after vaping with no significant changes in ventilation identified. Subsequent analysis showed that this change only occurred in participants who used nicotine‐containing e‐liquids. Paradoxically, they found a decrease in perfusion in smoking participants after smoking (Nyilas et al., 2022). These studies have shown inconsistent outcomes which may be a result of small sample sizes and the use of variable vaping products. In addition, both studies included relatively high EC exposures, with participants vaping for a period of 5–10 min (~10–20 puffs), outside of the MRI machine in an upright posture (Kizhakke Puliyakote et al., 2021; Nyilas et al., 2022).

In this study, we aimed to answer the following questions: (1) Does a typical single use of an EC (vape exposure 3–5 puffs), cause a measurable change in ventilation or perfusion? (2) Does nicotine concentration of the e‐liquid used, or (3) a history of smoking conventional cigarettes, or (4) sex‐differences have an impact on any changes observed in ventilation or perfusion? (5) Do any changes observed in ventilation or perfusion correlate with lung function metrics? In this study, we used oxygen‐enhanced MRI and Arterial Spin Labelling MRI methods to measure regional specific ventilation (SV) and lung perfusion (*Q*), respectively, in vaping volunteers before and immediately after vaping and also compared with a control cohort of never‐vaping/never‐smoking volunteers. Baseline spirometry and impulse oscillometry (IOS) measurements were obtained for all participants.

## 2 METHODS

### 2.1 Study and participant overview

**FIGURE 1:**
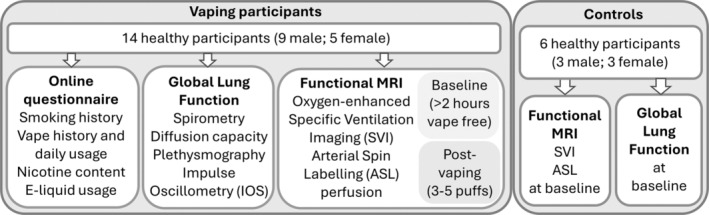
Study overview. Note, in the vaping cohort, global lung function was obtained at baseline (one time point only) and functional MRI was obtained at baseline (pre‐vaping) and (post‐vaping).

We measured regional specific ventilation (SV), pulmonary perfusion (*Q*), and tissue density using MRI in a cohort of 14 healthy vaping volunteers (9 male; 5 female) before and immediately after a single typical vape (generally 3–5 puffs of their regular vape). We recruited a total of 20 vaping participants based on the budgetary resources available with 14 acceptable datasets obtained. Vaping participants who were smokefree for at least 6 months prior to the study with no known cardiorespiratory conditions were recruited from within the New Zealand community. This study was approved by the University of Auckland Human Participants Ethics Committee (UAHPEC, Reference Number 023695). All subjects provided written informed consent prior to participation in the study. Participants attended two appointments, one where they underwent pulmonary function testing and the other for a 1‐hour MRI session. In addition, participants completed an online questionnaire to gather information on their vaping usage (history of usage, daily vaping frequency, nicotine strength, e‐liquid flavor, vape device and e‐liquid usage), and any history of smoking. Six healthy control subjects (never‐smokers, never‐vapers) were also recruited (UAHPEC, reference number 25350), and the same methods applied. An overview of the study is presented in Figure 1.

### 2.2 Lung function tests

Pulmonary function tests (PFTs), including spirometry, plethysmography, and IOS, were obtained on all participants according to current reference standards for normal healthy individuals (Quanjer et al., 2012). Measurements included resting lung volumes (via body plethysmography: total lung capacity, TLC; functional residual capacity, FRC; airway resistance, Raw; specific airway resistance, sRaw—calculated as the ratio of Raw multiplied by FRC (Criée et al., 2011)), forced spirometry (forced expiratory volume in 1 second, FEV1; forced vital capacity, FVC; peak expiratory flow, PEF), and diffusing capacity for carbon monoxide (DLCO). In all but three cases the MRI session preceded the PFT measurements. Most subjects were acquired both sets of data within a 2‐week period. However, in some cases the COVID‐19 pandemic resulted in delays such that three participants had a gap between sessions of 2–3 months, and two had a 5–6‐month gap (the time between MRI and PFTs for each participant are listed in the Appendix, Table A1).

IOS, a variant of the forced oscillation technique, uses variable frequency sound waves applied at the mouth during tidal breathing to provide a measure of airways resistance (*R*) and reactance (*Z*). The different frequencies can probe different depths within the airway tree, with the derived resistance at 5 Hz (R5) − resistance at 20 Hz (R20), showing the frequency dependence of resistance, providing a measure of small airway resistance (Foy et al., 2019). The resonant frequency (Fres) is defined as the frequency at which the inertial properties of airway and the capacitance of lung periphery are equal, with normal values in adults being 7–12 Hz (Desiraju & Agrawal, 2016). Area of reactance (AX) is the area under the reactance curve from lowest frequency to Fres. In both obstructive and restrictive lung disease involving the lung periphery, reactance, Fres, and AX have all been shown to increase (Desiraju & Agrawal, 2016). While IOS is not a standard clinical measure it has been shown to detect early dysfunction of the peripheral airways and may detect airway obstruction earlier than spirometry (Galant et al., 2017).

### 2.3 Imaging methods

#### 2.3.1 Image acquisition overview

MRI data were acquired using a 1.5 T Magnetom AvantoFit Siemens scanner (Siemens Healthineers, Erlangen, Germany). Imaging methods were implemented, based on previously published methods (Bolar et al., 2006; Henderson et al., 2009; Kizhakke Puliyakote et al., 2021; Sá et al., 2010, 2014; Theilmann et al., 2009), to measure specific ventilation (*SV*), lung perfusion (*Q*), and tissue density with minor adaptations to suit the Siemens scanner. Each participant was required to lie supine in the MRI scanner for approximately 45 min for vaping participants and ~ 25 min for controls. Subjects were fitted with a silicone facemask (Hans Rudolph, Shawnee, KS) attached to a flow bypass device to supply medical oxygen and room air, required for the specific ventilation imaging, as presented by Cook et al. (2015). Silicone phantoms were placed on the chest of the participant to provide reference standards for absolute quantification of perfusion and lung density (Holverda et al., 2011). A Biomatrix Body 18, 18‐channel torso coil (Siemens Healthineers, Erlangen) was placed on the subject's chest and used in conjunction with the in‐table spine coil array for image acquisition. A single 15 mm sagittal slice was acquired at the midpoint of the right lung, avoiding the major hilar blood vessels. Data were acquired at functional residual capacity (FRC, resting end‐expiratory lung volume). Lung tissue density scans were performed using both the body coil built into the scanner and the torso coil, allowing for construction of a coil sensitivity profile for each subject, which was used to correct for coil inhomogeneity in perfusion images, occurring in proximity to the coil elements, described in (Hopkins et al., 2007).

#### 2.3.2 Vaping exposure

The full MRI protocol, describe below, was obtained at baseline (pre‐vaping). Participants were asked not to vape for at least 4 h prior to the study. After the baseline images were acquired, vaping participants were removed from the MRI but remained on the MRI table in the supine posture for vape exposure. Most vape devices will contain magnetic metals and therefore are not MRI safe. To enable vaping within the MRI room, the MRI table was disconnected from the scanner and moved into the corner of the MRI room outside the 5 Gauss line. The vape device was carried into the scanner room staying as far away from the MRI machine as possible. Participants performed a typical vape session for them (ranging from 3 to 5 puffs) using their standard vape device and e‐liquid. Vaping participants were then maneuvered immediately back into the MRI scanner and the post‐vaping images acquired. The offset from the spine was used as a reference to ensure the same slice locations for the baseline (pre‐vaping) and post‐vaping scans. The pre‐ and post‐vaping images were not compared on a voxel‐by‐voxel basis. Instead, we compared the mean values and the relative dispersion measures of the full sagittal slice pre‐ and post‐imaging therefore any lack of alignment, which we expect to be minimal, will not impact on our results.

#### 2.3.3 Specific ventilation imaging

The method of Sá et al., previously described in detail (Prisk et al., 2020; Sá et al., 2010, 2014), was used. This method uses oxygen (O_2_) as a contrast agent to measure regional specific ventilation (SV) via the impact it has on the longitudinal relaxation time (*T1*) in lung tissue. SV is defined as the change in regional volume divided by the initial (end expiration) volume ($∆V/V_{0}$). SV is calculated using the change in image‐based signal intensity change over time on a voxel‐by‐voxel basis. This is based on the theory that after a sudden change in inspired fraction of O_2_, the rate of change of the alveolar O_2_ concentration is a function of the local SV. Lung units with higher SV reach a new equilibrium faster than units with a lower SV. Therefore, the time it takes to reach a new equilibrium is a measure of the local SV. This theory and the methodology used to derive the SV measure from MRI is described in detail in Sá et al. (2010). In brief, O_2_ was delivered via the facemask at a flow rate of ~100 L/min to exceed the maximal inspiratory flow rate. Subjects alternated between breathing room air (~21% O_2_) and 100% oxygen (O_2_) in 20 breath cycles (repeated three times) while images were acquired during the end‐expiration pause after every breath, at a rate of 10–12 breaths/minute (rate based on participant preference). O_2_ supply was switched during subject expiration using the actuation of a three‐way valve (Cook et al., 2015), to produce a stepwise change in inspired O_2_ concentration. An additional 20 breaths were added to the first 100% O_2_ cycle, to increase the ability to quantify slower equilibrating regions of the lung, requiring a total of 140 breaths (images) to complete the protocol.

Images were acquired with an inversion recovery prepared half‐Fourier single‐shot turbo spin echo (HASTE) acquisition with imaging parameters repetition time (TR) = 5000–6000 ms, echo time (TE) = 26 ms, field of view = 40 cm, receiver bandwidth = 850 Hz/px, and a full acquisition matrix of 256 × 128. The images were used to determine the rate of change of regional O_2_ indicating the rate at which resident gas within the lung is replaced by inhaled gas, governed by SV. All 140 MR images were registered and the time course of the signal for each image voxel was used to derive SV for the corresponding voxel. The mathematical methods used to characterize the SV based on the signal intensity information is described in Sá et al. (2010) and has been used in several other studies, i.e. (Henderson et al., 2013; Kizhakke Puliyakote et al., 2021; Prisk et al., 2020; Sá et al., 2014).

#### 2.3.4 Lung tissue density

Lung density was determined in sagittal slices acquired using a dual‐echo fast low‐angle shot (FLASH) sequence. This technique has previously been described and validated (Theilmann et al., 2009). This imaging sequence determines the voxel‐wise relative proton density by using the signal intensity at the two echo times to determine the local decay rate of the signal (T2*) and back‐extrapolates the signal to a time point of zero on a voxel‐by‐voxel basis assuming a monoexponential decay. Imaging parameters were: TR = 10 ms, TE = 0.82 ms and 1.8 ms, flip angle = 10°, slice thickness = 15 mm, field of view = 40 cm, receiver bandwidth = 1500 Hz/px, and a full acquisition matrix of 64 × 64. Five contiguous image slices were acquired to cover the whole lung, but only the centre slice, at the same location as the SV and ASL images, was used. The relative proton density was calibrated by the signal derived from the phantom (within the field of view and of known signal characteristics), to obtain regional lung proton (water) density in units of milliliter H_2_O per cubic centimeter lung, subsequently referred to as lung density. Density images were acquired with both the torso coil and the body coil.

#### 2.3.5 Pulmonary perfusion

Regional pulmonary perfusion was measured using a two‐dimensional (2D) arterial spin labelling (ASL) with a flow sensitive alternating inversion recovery with an extra radiofrequency pulse (FAIRER) imaging sequence and a half‐Fourier acquisition single‐shot turbo spin‐echo (HASTE) data collection schema, previously described (Bolar et al., 2006; Henderson et al., 2009). Two ECG‐gated images were acquired at mid‐diastole following application of an inversion pulse and a delay of 80% of one R–R interval (individually set for each subject): one using a spatially selective inversion pulse and the other using a spatially nonselective inversion. The two images were subtracted, and after correction for coil heterogeneity, a measure of blood delivered to the imaged slice during the preceding cardiac cycle was quantified (Bolar et al., 2006; Henderson et al., 2009). Imaging sequence parameters were as follows: inversion time (TI) = 560–920 ms (based on subject's heart rate), TR = 5000 ms TE = 18 ms, receive bandwidth = 900 Hz/px, field of view = 40 cm, slice thickness = 15 mm. The collected image matrix size was 192 × 96 (reconstructed to 192 × 192). Large vessels were filtered out to give a better estimate of perfusion using a threshold of 35% of maximum blood delivered based on previous modeling validation studies (Burrowes et al., 2012).

#### 2.3.6 Image analysis

Image analysis was performed using custom software written in MATLAB (Mathworks, Natick, MA). Relative dispersion (RD) was calculated as the ratio of spatial standard deviation over spatial mean for each of the SV and *Q* images as a measure of heterogeneity. SV and *Q* metrics extracted from the imaging (mean and heterogeneity) were compared in the vaping cohort before and after vaping and the vaping cohort was compared against the control cohort.

#### 2.3.7 Derivation of lung clearance index metric

An image‐based lung clearance index (LCI) was calculated from the SV map. LCI is the most commonly reported outcome measurement from the multiple breath washout test and provides a measure of ventilation heterogeneity. LCI represents the number of lung volume turnovers (FRCs) required to clear the tracer gas. To calculate LCI from the image data, a histogram of the SV values was created using all the voxels present in the SV map. Each SV value results in a mono‐exponential decay curve of gas concentration as a function of breath number, equivalent to a simulated multiple breath washout curve. A composite “whole lung” washout curve was then constructed as the sum of each image voxel washout, weighted by the frequency (the number of voxels with that SV) present in the histogram. The number of lung turnovers required to reach 1/40th of the initial simulated gas concentration was determined and converted to an image‐based LCI using the observed FRC. We propose that this image‐based LCI metric is likely to have some characteristics similar to LCI as derived from a multiple breath washout test, however the relationship between this metric and true LCI measurements remains to be evaluated. In this study, we assume that the SV map of the single slice imaged is representative of the overall lung.

#### 2.3.8 Statistical analysis

Data were compared within the vaping cohort before and after vaping using paired *t*‐tests. The vaping cohort data (before and after vaping) was also compared with the control cohort (at the single time point) using independent *t*‐tests. Spearman correlation analysis was performed across all measured variables, including participant demographics, lung function metrics, and image‐derived measurements. Analysis was performed to determine whether there was any influence of sex, nicotine concentration or type (freebase nicotine versus nicotine salt e‐liquid), and Body Mass Index (BMI). This analysis included correlation analysis, where appropriate, or splitting data into two groups (sex: male versus female; Nicotine: low, ≤20 mg/mL, versus high, >20 mg/mL, nicotine, freebase nicotine versus nicotine salt; low BMI ≤25 kg/m^2^ versus high BMI, >25 kg/m^2^) to determine whether there were differences across groups. These groups were compared using two‐tailed independent *t*‐tests. Levene's test was applied to determine whether the assumption of equal variances (*p* > 0.05) could be applied or not. All statistical analysis was performed using the IBM SPSS package. A *p*‐value of <0.05 was considered significant.

## 3 RESULTS

### 3.1 Cohort information and lung function

Key metrics collected in the online survey for each participant in the study demonstrating vaping usage (duration and frequency), nicotine levels, and smoking history are included in the Appendix in Table A1. All control (*N* = 6), and most vaping participants (*N* = 12), had spirometry and lung volume measures within normal healthy ranges (Table 1). FEV1/FVC in the two remaining vaping participants indicated mild impairment with *z*‐scores below the lower limit of normal (*z*‐score < −1.645; participant 12: *z*‐score = −1.67; participant 16: *z*‐score = −1.90). There were no significant differences in demographics (age, height, weight, BMI) between the vaping and control cohorts (Table 1).

**TABLE 1:** Subject demographics and pulmonary function tests in the vaping and control cohorts.

	Vaping cohort	Control cohort	*p*‐value
*N* (male/female)	14 (9/6)	6 (3/3)	
Age (years)	26.4 (8.0)	27.3 (3.0)	0.779
Height (cm)	173.8 (12.2)	174.8 (5.2)	0.844
Weight (kg)	74.4 (15.6)	73.1 (8.8)	0.845
BMI (kg/m^2^)	24.6 (4.0)	23.7 (2.2)	0.619
FVC (L)	5.5 (1.1)	5.2 (0.8)	0.620
FEV1 (L)	4.4 (0.9)	4.4 (0.7)	0.952
FEV1, %pred	109.7 (13.6)	109.5 (10.5)	0.781
FEV1/FVC (%)	80.8 (5.4)	84.3 (2.2)	0.051
FEV1/FVC, %pred	95.4 (5.8)	99.7 (4.1)	0.123
PEF, %pred	106.2 (7.1) *N* = 12	116.5 (10.4) *N* = 4	0.040*
TLC, %pred	103.2 (9.7)	102.6 (7.6)	0.884
FRC %pred	97.5 (15.3)	105.2 (13.5)	0.303
Raw (kPA/(L/s))	0.5 (0.1)	0.2 (0.1)	<0.001**
sRaw (kPA*s)	1.5 (0.3)	0.8 (0.2)	<0.001**
DLCO, %pred	101.6 (13.0)	109.5 (11.4)	0.216
R5 (Hz)	0.3 (0.1)	0.3 (0.1)	0.063
X5 (Hz)	−0.1 (0.0)	−0.1 (0.0)	0.504
Fres (Hz)	12.7 (3.6)	9.9 (2.0)	0.092
AX (kPA/L)	0.3 (0.3)	0.2 (0.2)	0.311
R5‐R20 (%)	17.1 (16.0)	4.8 (6.1)	0.023*
VT (L)	1.3 (0.7)	0.7 (0.2)	0.074

*Note*: All values are presented as means (SD). *p*‐values were determined using independent *t*‐tests with values <0.05 considered statistically significant ‐ indicated with * or with ** if *p* < 0.001. NB/ For PEF, % predicted values are only available for Caucasian participants (two missing values in Control cohort, and two missing in the vaping cohort). R5 (Respiratory resistance at 5 Hz), X5 (Reactance at 5 Hz), FRes (Resonant frequency); AX (Integrated area of low‐frequency reactance), R5‐R20 (%) is calculated as 100 x (R5‐R20)/R5—the % change in resistance from 5 Hz and 20 Hz.

Abbreviations: % pred, percent of the predicted value; BMI, body mass index; FEV1, forced expiratory volume in one second; FRC, functional residual capacity; FVC, forced vital capacity; PEF, peak expiratory flow; Raw, airway resistance; sRaw, specific airway resistance; TLC, total lung capacity; VT, tidal volume.

Peak expiratory flow (*PEF*), % predicted, was above 100% in both groups, but was significantly lower in the vaping cohort (*p* = 0.040). There was a significant elevation in airway resistance metrics in the vaping cohort compared to the control cohort, including airway resistance (*Raw*), specific airway resistance (*sRaw*), and *R5‐R20*, the difference between the resistance at 5 Hz and 20 Hz. Pearson correlation analysis (including all participants from both vaping and control cohorts, *N* = 20) showed that most of the metrics of pulmonary function described here were well correlated: FEV1/FVC correlated with sRAW (*R* = −0.495, *p* = −0.026); PEF correlated with Raw (*R* = −0.463, *p* = 0.040); PEF, % predicted correlated with sRaw (*R* = −0.677, *p* = 0.004); and R5‐20 correlated with Raw (*R* = 0.475, *p* = 0.034).

### 3.2 Regional perfusion and ventilation from MRI

**FIGURE 2:**
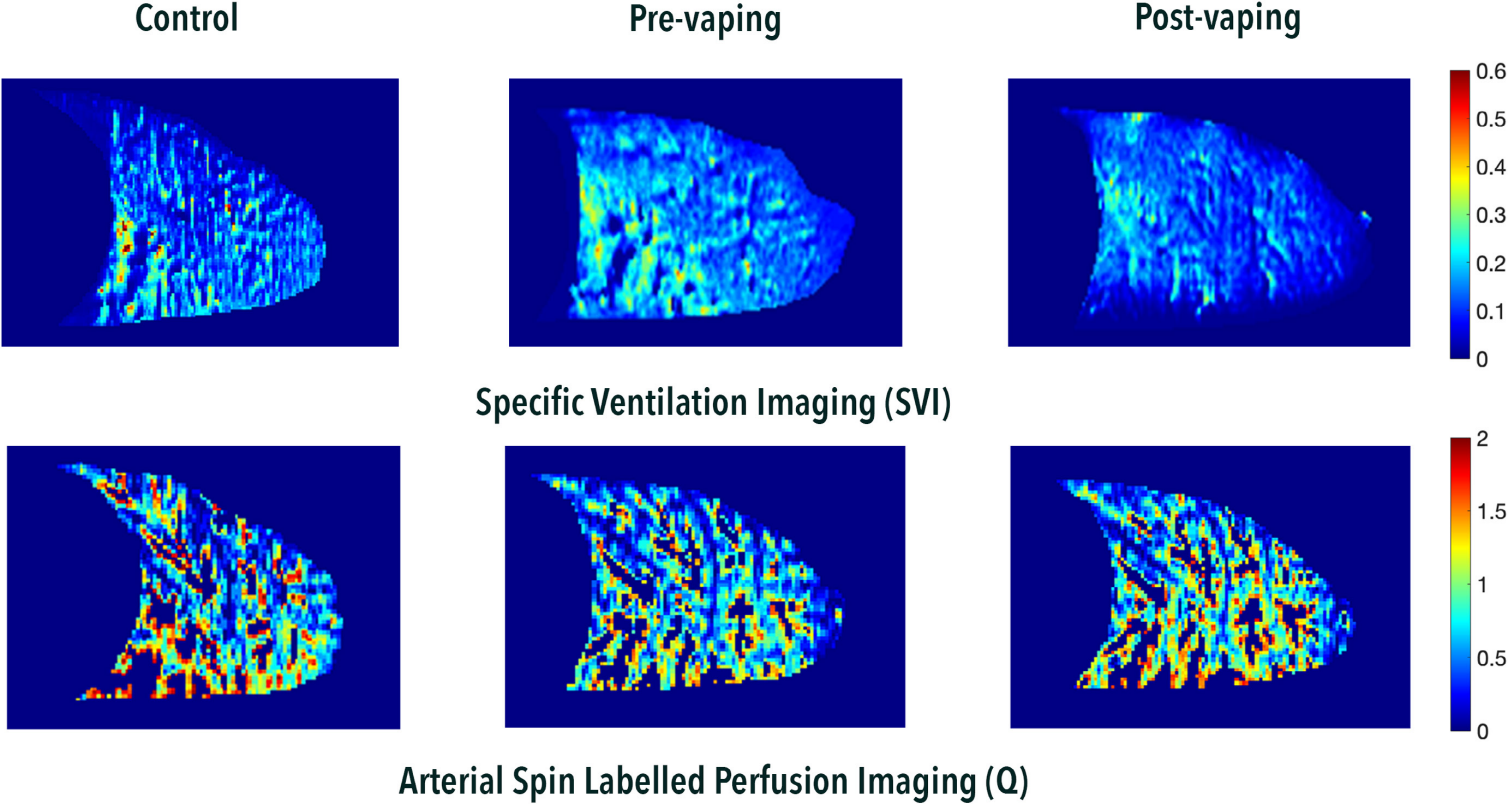
Example supine MR images in one control and one vaping participant (participant 19). Top panel demonstrates the processed specific ventilation (SV) image for control (left: Mean SV = 0.10, RD = 0.45) pre‐ (middle: Mean SV = 0.13, RD = 0.32) and post‐vaping (right: Mean SV = 0.10, RD = 0.45) whereby color scale represents SV values for each voxel as indicated by the spectrum on the right. Lower panel demonstrates the perfusion (*Q*) values extracted from the Arterial Spin Labelling imaging. Control (left: Mean *Q* = 0.73, RD = 0.78), pre‐vaping (middle: Mean *Q* = 0.67, RD = 0.68), post‐vaping (right: Mean *Q* = 0.83, RD = 0.56) and the color spectrums (far right). Missing voxels within the lung region of interest represent conduit vessels delivering blood to other lung regions that were removed using a threshold analysis (Burrowes et al., 2012). Voxels are not removed from the SV image. This is because SV is derived from 140 images and small variations is lung volume result in a smearing of the data such that the effective resolution in‐plane is ~1 cm. Thus, regions of the SV map corresponding to large vessels in the *Q* map represent lung tissue adjacent to the large vessels. While we could remove those voxels, the net effect on overall SVI parameters is small across all datasets (3.2% difference for mean SV and 1.0% difference for the RD of SV).

An example dataset showing specific ventilation and pulmonary perfusion (in a sagittal slice of the right lung) for a single vaping participant (participant 19) pre‐ and post‐vaping is demonstrated in Figure 2. In this participant, pulmonary perfusion (*Q*) increased post‐vaping and SV decreased a small amount after vaping. The change in mean *Q* and mean SV, and the change in relative dispersion of each measure, after vaping is included for each participant in the Appendix, Table A1.

Mean values of specific ventilation (SV) were 0.20 ± 0.07 and 0.15 ± 0.05 in the vaping (pre‐vaping) and control cohorts, respectively (Table 2). Values showing the change in SV and perfusion for each participant are included in the Appendix in Table A1. Neither the mean SV nor the relative dispersion (RD) of the SV were different between the vaping (pre‐vaping) and control cohorts, however immediately post‐vaping, the vaping cohort had a mean SV higher than the control cohort (*p* = 0.049, Table 2).

**TABLE 2:** Comparison of MRI metrics for vaping cohort (pre‐vaping) and control cohort.

	Control cohort	Vaping cohort (pre‐vaping)	Vaping cohort (post‐vaping)	*p*‐value (controls vs. pre‐vaping)	*p*‐value (controls vs. post‐vaping)	*p*‐value (vapers pre‐ vs. post‐vaping)
Mean SV	0.15 (0.05)	0.20 (0.07)	0.21 (0.06)	0.141	0.049*	0.596
RD SV	0.31 (0.15)	0.31 (0.12)	0.30 (0.13)	0.903	0.779	0.809
Image‐based LCI	5.02 (1.49)	6.33 (4.36)	6.05 (2.80)	0.487	0.407	0.849
Mean *Q* (mL/min/mL)	1.40 (0.60)	1.46 (0.76)	1.45 (0.68)	0.862	0.864	0.934
RD *Q*	0.58 (0.12)	0.62 (0.16)	0.60 (0.12)	0.588	0.768	0.440
Heart rate (bpm)	N/A	68.1 (10.5)	71.3 (8.7)	N/A	N/A	0.020*

*Note*: Post‐vaping values are compared with the same Control dataset which was only collected at one time point.

Abbreviations: LCI, lung clearance index estimated using the SV data; *Q*, perfusion determined from the arterial spin labelling MRI; RD, relative dispersion; SV, specific ventilation.

* *p* < 0.05.

Mean pulmonary perfusion (*Q*) values were not significantly different between the control and vaping cohorts, 1.40 mL/min/mL (range: 0.73–2.42 mL/min/mL) and 1.46 mL/min/mL (range 0.67–3.06 mL/min/mL), respectively (*p* = 0.862; Table 2). Mean *Q* did not change significantly post‐vaping (mean: 1.45 mL/min/mL; range: 0.65–3.15 mL/min/mL, *p* = 0.934; Table 2). No change in the relative dispersion (RD) of *Q* was observed between the control and vaping cohorts (*p* = 0.588; Table 2) or post‐vaping (*p* = 0.440; Table 2). The only metric that changed post‐vaping was heart rate, which increased significantly after vaping (*p* = 0.02, Table 2).

### 3.3 Correlation and grouped statistical analysis

#### 3.3.1 MRI metrics

A negative correlation existed between the pre‐vaping heterogeneity (RD) of SV and the change in the heterogeneity of *Q* post‐vaping (*R* = −0.672, *p* = 0.009). No other correlations were found.

#### 3.3.2 Sex

There were no statistically significant differences in the change in MRI metrics after vaping between males (*N* = 9) and females (*N* = 5), although the mean nicotine concentration used by males was higher than that used by females (male: 27.7 mg/mL versus female: 12.7 mg/mL, *p* = 0.043). Heterogeneity in perfusion was significantly different with higher heterogeneity in males (pre‐vaping: 0.66 versus 0.54 in females, *p* = 0.050; post‐vaping: 0.64 versus 0.53 in females, *p* = 0.016). No differences were observed in any of the other metrics between males and females.

#### 3.3.3 Nicotine

There was no correlation with the nicotine content used for each participant or any of the MRI metrics. In addition, there were no statistically significant differences when comparing those who vaped low levels of nicotine (classified as ≤20 mg/mL, *N* = 7) and high levels of nicotine (>20 mg/mL, *N* = 7). When categorized into those who used freebase nicotine liquids (*N* = 5) and nicotine salt e‐liquids (*N* = 9) no differences between groups were observed in any of the MRI or lung function metrics.

#### 3.3.4 BMI

**FIGURE 3:**
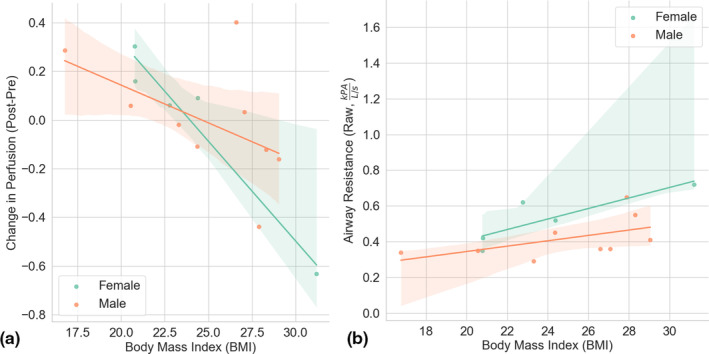
Significant correlations were found between the change in perfusion post‐vaping and BMI (a) and the airway resistance with BMI (b). The shaded regions colored for males and females display the 95% confidence interval.

There was a significant correlation (*p* = 0.003) between BMI and change in perfusion post‐vaping (Figure 3a) and with airway resistance measured via plethysmography and BMI (*R* = 0.615, *p*‐value = 0.019, Figure 3b). There was a strong negative correlation between BMI and FRC, % predicted (*R* = −0.829, *p*‐value = <0.0001) indicating that those with higher BMI were breathing at a lower lung volume. Airway resistance was also consistently higher in females (Figure 3b) as expected due to sex‐differences in airway anatomy.

**FIGURE 4:**
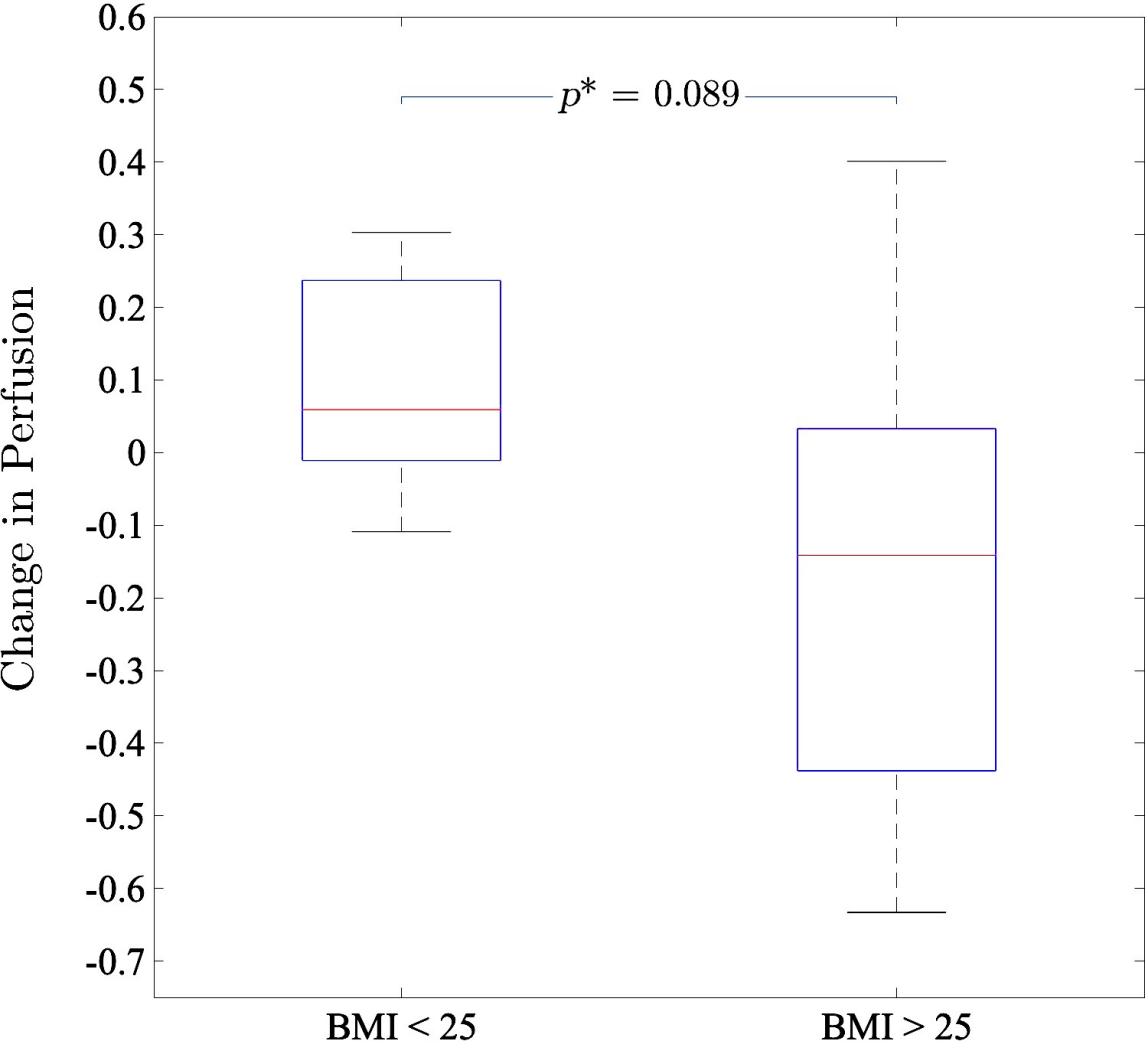
Change in perfusion (mL/min/mL) separated into high (BMI >25 kg/m^2^, *N* = 6) and low/normal (BMI ≤25 kg/m^2^, *N* = 8) BMI groups.

Comparison between a high BMI vaping group (BMI >25 kg/m^2^, *N* = 6) and low/normal BMI vaping group (BMI ≤25 kg/m^2^, *N* = 8) showed that, on average, those with a low/normal BMI had an increase in perfusion after vaping (mean change in *Q* = +0.1 mL/min/mL or + 10%), while those with a higher BMI had on average a reduction in blood flow (mean change in *Q* = −0.15 mL/min/mL or − 2%), although this did not reach statistical significance (*p* = 0.089, Figure 4).

#### 3.3.5 Previous smokers versus never‐smokers

Half of the vaping cohort (*N* = 7) had a prior history of smoking conventional cigarettes with a mean pack‐year of 5 (range: 1.5–10 pack‐years, refer to Table A1). There were no differences in any of the measured variables between those who had a previous history of smoking and never smokers.

## 4 DISCUSSION

MRI measurements of specific ventilation (SV) and perfusion (*Q*) showed no statistically significant differences between never‐vaping/never‐smoking Controls and an asymptomatic vaping cohort. Metrics extracted from MR images in the vaping cohort before and immediately after 3–5 puffs of vape exposure did not show any significant changes in measured lung function after vaping. However, there were significant differences in the non‐imaging lung function measurements between the vaping and control cohorts particularly in measurements representative of small airways resistance, indicating increased airways resistance in the vaping cohort. While we did not see acute changes in regional lung function (measured via MRI) after vaping, the vaping cohort showed signs of changes in lung function from chronic vape exposure (as measured by pulmonary function tests).

Our study showed a significant increase in heart rate after vaping. This is not a new finding, with several previous studies having demonstrated this (i.e. (Kizhakke Puliyakote et al., 2021; McClelland et al., 2021; Vansickel & Eissenberg, 2013)). This is due to nicotine's effect on cardiac sympathetic nerve activity (Moheimani et al., 2017) and via adrenergic mechanisms (Centers for Disease Control and Prevention (US), 2010); however, animal studies have shown that e‐cigarette solvents alone (propylene glycol, vegetable glycerol) can induce changes in heart function (heart rate and heart rate variability) (Carll et al., 2022). Participants used a range of nicotine concentrations; however, we did not find any differences in our measurements as a function of nicotine levels. Nicotine salt products are typically of higher nicotine concentrations and are more easily absorbed into the bloodstream (Gholap et al., 2020). Despite this, we did not see any differences due to the type of nicotine product (freebase nicotine compared to nicotine salt products) that participants used.

Half of the vaping cohort had a history of smoking conventional cigarettes; we did not see any evidence of differences in any lung function in those who had previously smoked compared to those who had not. There may be several reasons for this. One is the relatively small sample sizes (7 in each of the previous‐ versus never‐smokers). Plus, the vaping cohort was relatively young, with an average age of 26 years old (standard deviation = 8 years; range: 18–50 years old) and had relatively low pack‐year exposure (mean pack‐year = 5; range 1.5–10 pack‐years).

There was a significant correlation between BMI and the change in perfusion after vaping. This was an unexpected finding; however, it is likely to be caused by the supine inhalation method of our vaping participants. Two previous MRI vaping studies, which have both shown an overall increase in blood flow after vaping, have removed participants from the MRI scanner/bed and participants have used their vape devices while standing (Kizhakke Puliyakote et al., 2021; Nyilas et al., 2022). In our study, vaping participants inhaled from their devices in the supine posture to ensure limited movement between image acquisitions and a rapid return to the scanner after vaping. Six of the eight participants with a normal/low BMI (≤ 25 kg/m^2^) had an increase in blood flow after vaping which is what we were expecting based on previous studies; on average low BMI participants had a 10% increase in mean perfusion after vaping. Four of the six participants with a higher BMI (> 25 kg/m^2^) had a reduction in blood flow after vaping; on average this group had a very small reduction in blood flow of 2% after vaping. The presence of adipose tissue around the rib cage and abdomen and in the visceral cavity loads the chest wall in overweight and obese people reduces functional residual capacity (FRC) (Salome et al., 2010). These fat deposits, and functioning at a lower FRC, reduce the compliance of the lungs, which results in slightly lower tidal volumes with increasing BMI (Dixon & Peters, 2018). These effects are exacerbated in the supine posture due to the redistribution of adipose tissue in this posture (Lo Mauro et al., 2023). This variation between BMI groups could indicate a smaller vape dose per unit body weight in the higher BMI group (due to the lower FRC volume) or could simply indicate that different people respond differently to the vaping chemicals.

A recent commentary by Hernandez Cordero and Sin (Hernández Cordero & Sin, 2024) recapped the fact that while smoking and air pollution are the main environmental contributors in the development of COPD, it is also known that low BMI, alongside these environmental factors, is a consistent and significant risk factor for rapid disease progression in patients with COPD (Berrington de Gonzalez et al., 2010; Sun et al., 2019). Compared to normal BMI (18–25 kg/m^2^), low BMI is associated with faster FEV1 decline and the reverse is true for high BMI (Sun et al., 2019). This finding has been termed the “obesity paradox”, whereby higher BMI appears to be protective of lung function, while the reverse is true for cardiovascular disease. The mechanisms for this are unknown and likely emerge due to multiple factors. Because lung function and BMI are both heritable traits, a recent study by Zhang et al. (2024) investigated the relationship between genetically predicted BMI with mortality (all‐cause, cardiovascular, and respiratory mortality) in patients with COPD. They showed that those with actual BMI higher than the genetically predicted BMI was associated with higher cardiovascular mortality but not respiratory mortality. Those with discordantly low BMI, compared to their genetically predicted BMI, had higher all‐cause and respiratory mortality compared to those with concordant (actual BMI was well matched with genetically predicted value) BMI. While these previous studies demonstrate the correlation, the mechanisms underlying this paradox remain unknown. Our results are consistent with this “obesity paradox” in that those with higher BMI did not show an increase in blood flow potentially indicating either a lower exposure from vaping or differences in the uptake to the bloodstream.

Participants in our study stated that a normal vaping session for them was between 3 and 5 puffs. We wanted to capture a typical vape session and therefore kept the exposures at this level. This level of exposure was lower than previous studies where participants took around 10–20 puffs (19 puffs ±13 puffs were vaped for 4.9 min ±1.1 (Nyilas et al., 2022); ~10 puffs over 5–10 min (Kizhakke Puliyakote et al., 2021)) and may be why we did not see significant changes after vaping across the vaping cohort as a whole. A post‐hoc power calculation showed that we would have required a sample size of >185,000 participants (with the mean pre‐ and post‐vaping perfusion values and corresponding standard deviations of this data from the current study). The very large sample size is a consequence of the extremely small effect size calculated (Cohen's *d* = 0.009). When the effect size is very small, a much larger sample size is needed to detect the effect with sufficient power. Since that n is very large, we are confident in our conclusion of no physiologically meaningful effect. Python library statsmodels was used to conduct the post‐hoc power calculation. A two‐sample *t*‐test was assumed. Cohen's d was used to calculate the effect size based on difference between the means of the two groups divided by the pooled standard deviation. The test assumed that both groups followed normal distribution. The significance level of 0.05 was used and the desired power of 0.80 was used.

The MRI methods applied in this study have been applied in several research studies, for example (Geier, Neuhart, et al., 2018; Henderson et al., 2013; Hopkins et al., 2007; Sá et al., 2017), including the vaping study of Kizhakke Puliyakote et al. (2021). There are some limitations of our study. The current acquisition method only enables SV and *Q* images to be obtained in a single slice of the right lung. We are assuming vaping has a global effect on lung, and thus this slice is representative of the lungs as a whole, but it is possible we are missing useful regional information from other parts of the lungs. Our sample size is small (although on par with other studies), consisting of 14 vaping participants and six non‐vaping controls which may not be representative of the population. Control participants did not undergo a “sham” exposure due to time and budgetary constraints which may have been useful to include as a comparison of the MRI protocol itself. This would have been useful to, for example, determine whether O_2_ administration affected the perfusion results pre‐ and post‐vaping. However, for this issue, there have been numerous SVI studies in which test–retest scans have been made using this combined SV (O_2_‐enhanced) and perfusion technique (Geier et al., 2019; Geier, Kubo, et al., 2018; Geier, Neuhart, et al., 2018; Sá et al., 2014). These have shown minimal changes therefore should not affect our results.

E‐cigarettes are extremely variable in their design, functionality and the e‐liquids used within them also vary widely. In this study, participants used their own standard vaping devices and e‐liquid and performed a vaping session (3–5 puffs) that was typical for them. This led to variability in the number of puffs taken in the study, the concentration and type of nicotine product used, the e‐liquid flavor, and potentially even the power settings and coil type used in the vaping device. The range of puffs used mean that there is nearly a 2‐fold difference in the vape exposure during the intervention. Participants were asked to take their ‘typical’ vape exposure, such that they could feel the effects of the vape/nicotine on their body. We did not find any correlation with the number of puffs taken and the outcomes measures extracted from MRI, however this lack of puff and device standardization could potentially impact on the conclusions made in our study. Little is known about whether these factors will influence the effect of vaping on the body. Finally, as demonstrated by the discussion above related to BMI, participants vaped during the study in the supine posture. This is likely an unnatural position for vaping and, especially in participants with higher BMI, may have led to smaller puff volumes during vaping.

There was no acute effect of a single “typical” vape exposure in these subjects; however, there was evidence of overall degradation in lung function in the vaping cohort compared to the control cohort, particularly in the peripheral airways. While asthma can impact the full airway tree, the peripheral or small airways are important and often the main site of airflow limitation. Key methods to measure peripheral airway function are via function imaging, impulse oscillometry, or the multiple breath washout technique (King et al., 2024). We did not see significant differences in mean specific ventilation, or the relative dispersion of this metric measured via MRI between the control and vaping cohorts, but we did see significant differences in some of the pulmonary function test measurements. The metrics that differed significantly were peak expiratory flow (*PEF*), % predicted, airway resistance (*Raw*), specific airway resistance (*sRaw*), and *R5‐R20* measured via IOS. These measures demonstrated an increase in airways resistance in the vaping cohort compared to the never‐vaping controls. R5‐R20 (%) is a measure of the frequency dependence of airways resistance and has been demonstrated by various studies to be a metric of peripheral airway function, or small airways resistance (Foy et al., 2019; King et al., 2024; van den Berge & Kerstjens, 2019). Two of the vaping participants had FEV1/FVC just below the lower limit of normal indicating early signs of airway obstruction. These measurements could indicate early signs of an asthma‐like phenotype in the vaping cohort, as suggested by other studies in the literature (Hickman et al., 2024; Pérez et al., 2024; Roh et al., 2023). Combined with other studies which used a greater vaping exposure than in this study, the results suggest that there is a cumulative negative consequence of vaping on lung function.

## ETHICS STATEMENT

This study was approved by the University of Auckland Human Participants Ethics Committee (UAHPEC, Reference Number 023695), for the vaping participants, and UAHPEC, reference number 25350, for the control participants.

## Data Availability

The data that support the findings of this study may be available on request from the corresponding author [KSB]. The data are not publicly available due to restrictions around the ethics approval for this study.

## APPENDIX A

### A.1

Table A1 presents key metrics collected in the online survey for each participant in the study demonstrating vaping usage (duration and frequency), nicotine levels, and smoking history. The MRI‐extracted functional information is also included for each participant representing the change in specific ventilation and perfusion after vaping and the change in relative dispersion of these measures post‐vaping.

**TABLE A1:** Online survey information captured for vaping participants and MRI extracted information representing the change (Δ) in specific ventilation (SV) post‐vaping, the Δ in perfusion after vaping and the Δ relative dispersion (RD) for both SV and perfusion for each participant.

Subject ID	Sex	Duration of vaping	Frequency of vaping	Nicotine strength	Nicotine type	Smoking history (pack years)	Time between PFT and MRI	Δ SV (post‐pre vape)	Δ RD SV (post‐pre vape)	Δ perfusion (post‐pre vape, mL/min/mL)	Δ RD perfusion (post‐pre vape)
6	F	1–2 years	10–20 times/day	Low	Salt	2.5	≤1 week	0.07	−0.16	0.09	−0.14
7	M	>2 years	>20 times/day	High	Freebase	10	≤1 week	−0.07	−0.26	−0.16	−0.18
8	M	6–12 months	>20 times/day	Medium	Salt	5	6 months	0.02	0.00	−0.11	0.07
9	M	1–6 months	>20 times/day	Medium	Freebase	0	5 months	0.02	−0.09	0.29	−0.11
10	M	1–2 years	Less than daily	Extra high	Salt	0	≤1 week	−0.01	0.02	−0.02	0.09
11	M	1–2 years	>20 times/day	High	Salt	0	≤1 week	0.01	−0.03	0.06	0.00
12	M	>2 years	>20 times/day	Extra high	Salt	3	≤1 week	−0.06	−0.04	−0.44	0.14
13	F	1–2 years	>20 times/day	Low	Freebase	5	≤1 week	0.01	0.20	−0.63	0.08
14	M	>2 years	>20 times/day	Extra high	Salt	1.5	≤2 weeks	0.06	0.07	0.40	−0.04
15	F	1–2 years	>20 times/day	Extra high	Freebase	0	≤1 week	0.14	−0.08	0.06	−0.01
16	M	>2 years	>20 times/day	Extra high	Salt	0	≤1 week	0.02	−0.27	−0.12	−0.05
18	M	>2 years	>20 times/day	Extra high	Salt	0	2 months	−0.06	0.31	0.03	−0.12
19	F	>2 years	>20 times/day	Low	Freebase	8	3 months	−0.03	0.13	0.16	−0.13
20	F	>2 years	>20 times/day	High	Salt	0	2 months	0	0.05	0.30	0.08

*Note*: Nicotine levels: Low: <8 mg/mL; Medium: 9–16 mg/mL; High: 17‐24 mg/L; Extra high: >24 mg/mL.

Abbreviations: PFT, Pulmonary Function Test; RD, relative dispersion; SVI, specific ventilation.
