# Correction to “Vaping causes an acute BMI‐dependent change in pulmonary blood flow”

**DOI:** 10.14814/phy2.71109

**Published:** 2026-09-22

Burrowes, K. S. , Seal, M. , Noorababaee, L. , Pontré, B. , Dubowitz, D. , Sá, R.C. , & Prisk, G.K. (2024). Vaping causes an acute BMI‐dependent change in pulmonary blood flow. Physiological Reports, 12, e70094. 10.14814/phy2.70094 PMC1148900039424421

In the author list, the name “L. Noorababaee” was incorrect. This should have read “L. Noroozbabaee”.

We apologize for this error.

